# Nail patella syndrome: a rare cause of renal failure in a young adult

**DOI:** 10.4314/pamj.v9i1.71207

**Published:** 2011-07-21

**Authors:** Nagendra Boopathy Senguttuvan, Arjun Sivaraman, Devasenathipathy Kandasamy, Kanniraj Marimuthu

**Affiliations:** 1 Department of Cardiology, All India Institute of Medical Sciences, New Delhi, India; 2 Department of Surgery, Kasturba Medical College, Manipal, India; 3 Department of Radiology, All India Institute of Medical Sciences, New Delhi, India; 4 Department of Orthopedics, All India Institute of Medical Sciences, AIIMS, New Delhi, India

**Keywords:** Renal failure, nail dysplasia, absent patella

## Abstract

Nail Patella Syndrome (NPS) is a rare hereditary disease affecting multiple systems with predominant involvement of Kidney, Bones and Nails and Eyes. We report a case of NPS which presented as renal failure in a 22 year old male. The patient was admitted with decreased urine output and features of fluid overload and was being evaluated for renal failure. Physical examination revealed associated bony deformities which raised the suspicion of NPS as a possible etiology. This was confirmed by the radiological evaluation which showed the classical features of NPS. Though NPS is a rare clinical condition, physicians should complete knowledge about the components of NPS for appropriate diagnosis and for early detection of other systems involvement.

## Introduction

Nail patella syndrome is a rare autosomal dominant hereditary disease with variable penetrance and expression. Bones and nails, eyes and kidneys are the systems commonly involved. Renal involvement is the most common and serious manifestation of the disease involving 40% of the affected individuals. LMX1B gene located in the chromosome 9 is considered to be responsible for the various manifestations of the disease. The management involves early identification of the systems involved and appropriate treatment.

## Case report

A 22 year old young male from Nepal was symptomatic for the 2 months with intermittent nausea, vomiting, loss of appetite and breathlessness. His symptoms increased progressively during past 10 days with facial puffiness, pedal edema, decreased urine output and breathlessness. He denied orthopnoea, PND and any symptoms suggestive of seizures, hematuria, pyuria, lithuria. There was no family history of similar illness. Ten years back he had a fall from height and fracture of spine for which an ORIF was done.

### Assessment

**Figure 1:**
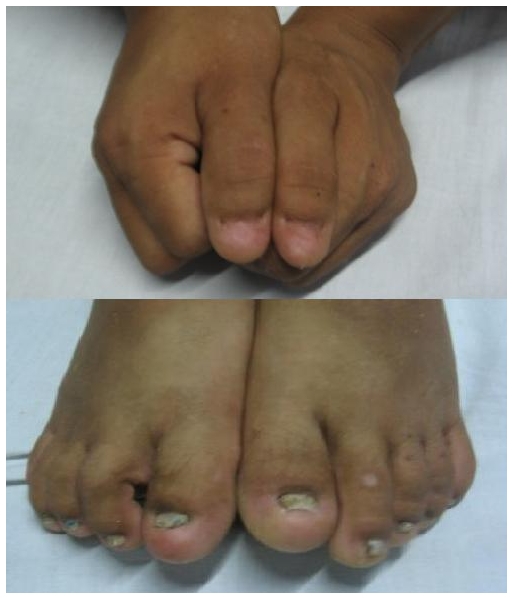
Clinical picture showing dysplastic nails in all digits

A physical examination performed revealed a pulse rate of 86/ minute with a blood pressure of 140/80 mm Hg. He was pale and his jugular venous pressure was elevated to around 7cm. There was bilateral pitting pedal edema. He had deformed nails in all 10 fingers and toes more prominent in the thumbs (Figure 1). He had deformed knee joints along with fixed flexion deformity of bilateral hip joints. Examination of his chest revealed fine crepitations in the basal region. His cardiovascular system examination showed normal heart sounds with functional systolic murmur in the pulmonary area with no pericardial rub. His abdominal and CNS examinations were normal. Fundus examination showed grade II hypertensive retinopathy.

**Figure 2:**
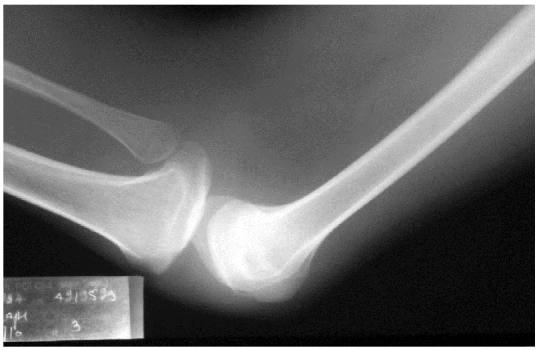
X-Ray of the knee showing absent of patella

**Figure 3:**
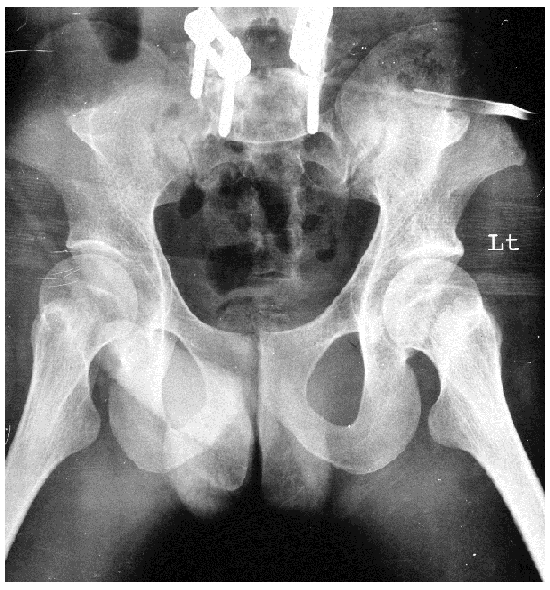
X-Ray of pelvis showing bilateral iliac horns

He was worked up for his renal failure. His urea and creatinine were 20 mg% and 28% respectively. His initial ABG showed metabolic acidosis. His 24 hr urine protein revealed nephrotic range (3 g/ day) of proteinuria with no active sediments in the urine routine examination. His USG KUB revealed right kidney of 9.9 cm with ill defined corticomedullary junction a non-visualized left Kidney (ectopic / absent). X-Ray of B/L knee joint showed dysplastic patellae (Figure 2) and X-Ray of the pelvis showed B/L iliac horn (Figure 3). A computerised tomogram of B/L kidneys was done which revealed the presence of both shrunken left and right kidneys (7 cm each). Considering his entire clinical picture consisting of nephrotic range proteinuria, azotemia, uraemia, dystrophic nails, and deformed knee joints with dysplastic patella with iliac horn in the X-ray of pelvis, Nail patella syndrome was diagnosed.

### Management

This young male was managed with renal replacement therapy (RRT) initially in the form peritoneal dialysis later followed by hemodialysis. After RRT, he showed symptomatic improvement. He is being managed conservatively as a patient with Nail patella syndrome with chronic renal failure on maintenance hemodialysis.

## Discussion

Nail-patella syndrome (NPS) (otherwise known as Fong′s Disease, Hereditary Onychoosteodysplasia (HOOD) or Turner-Kieser Syndrome) is an autosomal dominant hereditary disorder related to ABO blood group. The gene responsible for nail- patella syndrome is located in chromosome 9q34 and is termed as LMX1B. This gene is responsible for the normal dorsoventral patterning of the limb and normal development of the glomerular basement membrane in the kidney [1]. Being an autosomal dominant disorder, the chance to have an affected child is 50% per pregnancy. Though the incidence of disease varies, it is roughly estimated to occur in 1 in 50,000 newborns.

Nail- Patella syndrome is usually characterized by nail dysplasia, patellar apoplasia/hypoplasia, iliac horns, elbow dysplasia, and frequently primary open angle glaucoma and progressive nephropathy. The phenotypic expression of the disease varies greatly between each individual due to variable penetrance.

### Nail-ostial manifestations

Most of patients with Nail Patella Syndrome have affected fingernails, which may be partly or completely missing (80-100% of cases). Thumbs are affected severely as compared to other fingers, and the severity decreases in severity toward the little finger. The crescent “moons” at the base of the nail are often triangular. The distal most joint which is actually closer to the nail may not have creases over it. Sometimes, fingers might seem to be double-jointed. Some of these patients with Nail Patella Syndrome are unable to fully straighten their joint. Tendon and ligaments may be misplaced, and webbing sometimes forms around the elbow. One of the characteristic manifestations of nail patella syndrome is aplasia (absence) or hypoplasia (underdeveloped) patella. If patella is present, it will be hypoplastic, and it may be located more distally than usual leading to recurrent dislocation. The hypoplastic patella may be ovoid, triangular or irregular in shape. Abnormal projections of bone from the superior (upper) portion of both sides of the hip bone (bilateral iliac horns) are reported. These exostoses (′horns") of the ilia are often evident. These "horns" are very specific to this disease, but are seen in only 30-70% of these patients. They may be visible, palpable or impalpable, depending on size. Other bony manifestations described are hypoplasia of the scapulae, talipes (club foot) or “twisted feet”, hypoplasia of the first ribs, dislocation of the head of the radius, prominent outer clavicle, malformed sternum (Pectus Excavatum), scoliosis, and lordosis. There is also aplasia of pectoralis minor, biceps, triceps and quadriceps.

### Kidney manifestations

The incidence of renal impairment in NPS is approximately 40% [2]. This is one of the most serious manifestations of NPS. Though it is common in adults, it has been described to occur in children also. The clinical manifestations include protienuria with or without hematuria, and hypertension. Nephrotic syndrome and progressive renal failure can occur. The glomerular changes by light microscopy are quite non-specific, varying in degrees of focal and segmented sclerosis and often segmented thickening of the capillary wall. Sometimes, the microscopy reveals a unique lesion consisting of a moth-eaten appearance of the glomerular basement membrane. This condition is said to be a generalised disorder of the mesenchyme. The course is rather benign with renal failure a late feature. Annual urine screening is recommended. Kidney biopsy is rarely required to diagnose the disease. If a transplant is deemed necessary, it should be noted that recurrence after transplant does not happen.

### Ophthalmological manifestations

It is not very late that researchers at the University of Michigan Health System have found strong evidence of a link between open angle glaucoma and NPS. Studies suggest indirect evidence that the genetic location of the two conditions may be located at the same chromosomal site. Based on this study, it was advisable to have eye check-up in these patients regularly. Other ocular abnormalities that have been reported are keratoconus, microcornea, microphakia, cataracts and ptosis

### Other manifestations

Dental Problems, hyperthyroidism, irritable bowel syndrome, tilted uterus, smaller stature, and cleft lip/palate are some manifestations that are considered to occur in these patients with NPS.

### Radiographic Features

**Pelvis:** Small iliac wings with bilateral horns (seen up to 70% of cases) arising from the central area of the iliac wings, directed posterolaterally, located at the site of attachment of gluteus medius muscles [3].

**Limbs:** Patellae may be absent or hypoplastic. They may be irregularly ossified or dislocated. Disproportionate prominence of medial condyle of femur may be seen. Radial head may show hypoplasia with lateral inclination of radial head which may be associated with radial subluxation or dislocation. Lateral humeral epicondyle and capitellum may be hypoplastic. Other features like clinodactyly, camptodactyly equinovalgus deformity of hind foot, calcaneovalgus deformity, forefoot supination and lateral subluxation of tarsal-metatarsal joints and ball-and-socket type of ankle joint may be seen [4].

**Chest:** Hypoplastic scapula with glenoid dysplasia, thickening and convexity of lateral border of scapula may be seen. A conical spine like projection may occasionally be seen in the lateral aspect of clavicle which is called “clavicular horn” [5].

**Joints:** Finger joints may show laxity with asymmetrical development of joint (hypoplasia or hyperplasia)

## Conclusion

The multi system involvement of NPS clearly indicates the need for the physicians of different specialties to have a thorough knowledge about the syndrome to appropriately identify the condition and also for the early detection of other system involvement to prevent the associated complications.
